# High Prevalence of Antibiotic-Resistant *Mycoplasma genitalium* in Nongonococcal Urethritis: The Need for Routine Testing and the Inadequacy of Current Treatment Options

**DOI:** 10.1093/cid/cit752

**Published:** 2013-11-26

**Authors:** Marcus J. Pond, Achyuta V. Nori, Adam A. Witney, Rose C. Lopeman, Philip D. Butcher, Syed Tariq Sadiq

**Affiliations:** 1 Centre for Infection and Immunity, St George's University of London; 2 Department of Genitourinary Medicine, St George's Healthcare NHS Trust, London, United Kingdom

**Keywords:** *Mycoplasma genitalium*, antimicrobial resistance, nongonococcal urethritis, sequence typing

## Abstract

***Background.*** Empirical antibiotic therapy for nongonococcal urethritis (NGU) and cervicitis is aimed at *Chlamydia trachomatis*, but *Mycoplasma genitalium*, which also commonly causes undiagnosed NGU, necessitates treatment with macrolides or fluoroquinolones rather than doxycycline, the preferred chlamydia treatment. Prevalence of *M. genitalium* and associated genotypic markers of macrolide and fluoroquinolone resistance among men symptomatic of urethritis were investigated. Genetic diversity of *M. genitalium* populations was determined to infer whether findings were applicable beyond our setting.

***Methods.*** *Mycoplasma genitalium* and other NGU pathogens were detected using nucleic acid amplification methods, and DNA sequencing was used to detect genotypic resistance markers of macrolide and fluoroquinolone antibiotics in 23S ribosomal RNA, *gyrA*, *gyrB*, and *parC* genes. MG191 single-nucleotide polymorphism typing and MG309 variable number tandem analysis were combined to assign a dual locus sequence type (DLST) to each positive sample.

***Results.*** Among 217 men, *M. genitalium* prevalence was 16.7% (95% confidence interval [CI], 9.5%–24.0%) and *C. trachomatis* prevalence was 14.7% (95% CI, 7.8%–21.6%) in NGU cases. Nine of 22 (41%; 95% CI, 20%–62%) patients with *M. genitalium* were infected with DLSTs possessing genotypic macrolide resistance and 1 patient was infected with a DLST having genotypic fluoroquinolone resistance. Typing assigned *M. genitalium* DLSTs to 2 major clusters, broadly distributed among previously typed international strains. Genotypic macrolide resistance was spread within these 2 clusters.

***Conclusions.*** *Mycoplasma genitalium* is a frequent undiagnosed cause of NGU in this population with rates of macrolide resistance higher than those previously documented. Current guidelines for routine testing and empirical treatment of NGU should be modified to reduce treatment failure of NGU and the development of further resistance.

## Abstract (precis)

*Mycoplasma genitalium* infections were as frequent a cause of nongonococcal urethritis as *Chlamydia trachomatis*, had high rates of macrolide-associated genotypic resistance, and were nonclonal, suggesting an established community infection. Detection of genotypic resistance to fluoroquinolones is cause for concern.

*Mycoplasma genitalium* and *Chlamydia trachomatis* are recognized causes of common genital tract morbidities, such as pelvic inflammatory disease and nongonococcal cervicitis in women [1] and nongonococcal urethritis (NGU) in men [2, 3]. UK- and US-based antimicrobial treatment guidelines for these syndromes are aimed at *Chlamydia* infection, which is more frequently detected and has a strong evidence base for causing chronic disease [4, 5], whereas *M. genitalium* is not usually recommended among organisms for routine testing in routine sexually transmitted infection screening. NGU, diagnosed using both clinical presentation with Gram-stained urethral smear microscopy, is empirically treated with a 1-week oral course of doxycycline or a single oral dose of 1 g of azithromycin [4, 5]. Both are effective against *C. trachomatis*, although clinical failure to eradicate *Chlamydia* with single-dose azithromycin has been reported [6]. For *M. genitalium*, doxycycline has poor efficacy, evidenced by high rates of clinical failure with tetracyclines [7]; treatment failure with single-dose azithromycin is increasingly evident and associated worldwide with strains possessing single-nucleotide polymorphisms (SNPs) in its ribosomal RNA gene (domain V, 23S rRNA) [8–10]. Moxifloxacin, a fluoroquinolone demonstrating efficacy against *M. genitalium*, is recommended following treatment failure with macrolides and tetracyclines [1]. Fluoroquinolone resistance–associated SNPs in gyrase (*gyrA*) or topoisomerase IV (*parC*) genes have been detected in *M. genitalium* from patients exhibiting fluoroquinolone treatment failure and phenotypic resistance in vitro [11].

In this study of men symptomatic of urethritis, we investigated the prevalence of *M. genitalium* in comparison to established causes of urethritis and determined frequencies of known genetic resistance markers to macrolides and fluoroquinolones within *M. genitalium*. We sought to establish whether prevalence of *M. genitalium* and resistance was local only to our population or could be applicable more generally, using a previously validated dual-locus typing system.

## METHODS

### Patients

This was an observational study of all men prospectively presenting with symptoms of urethritis to the Genitourinary Medicine Clinic at St George's Hospital, Tooting, London, between 28 September and 15 December 2011. This study was conducted with the approval of the South West London Research Ethics Committee (number Q0803 71). Patients were designated as having urethritis, NGU, nonchlamydial NGU, or no urethritis. “Urethritis” was defined as any patient receiving treatment specifically for urethritis in that clinical episode and at least 1 of the following: (1) recent history of dysuria, urethral discomfort, or urethral discharge, and visible urethral discharge on examination; (2) ≥5 neutrophils per high-power field on urethral Gram stain. NGU was defined as urethritis where gonorrhea was excluded by at least 2 of Gram stain, nucleic acid amplification test (NAAT), and routine gonococcal culture. Nonchlamydial NGU was defined as NGU where chlamydia had been excluded by NAAT. Only patients consenting to routine urethral smears were included in the study. Patients had a urethral smear prepared with a cotton-tipped swab or plastic loop and were asked to provide a maximum of 50 mL first-catch urine specimen. Clinical notes of patients with urethritis were reviewed at 3 months after presentation to assess instances of treatment failure.

### Pathogen Detection

First-void urine samples were collected routinely for chlamydia and gonorrhea NAATs using the Viper system (Becton Dickinson, Oxford, UK). The residual of these samples were retrieved and used for detection of *M. genitalium* and *T. vaginalis* using primers as previously described [12, 13]. DNA extracts positive for *M. genitalium* were investigated for SNPs in the quinolone resistance–determining regions (QRDRs) of *gyrA*, *gyrB*, and *parC* and macrolide resistance SNPs in 23S rRNA [8] and also typed using a multiple locus variable number tandem repeat marker in MG309 (MG-309-STR) and *mgpB* SNP analysis [14]. Further details of the protocol are provided in Supplementary Data 1.

### Statistical Analysis

To detect the difference of 25% and 4% of *M. genitalium* in patients with and without urethritis, respectively [1], with 80% power and α = .05, 111 urethritis and nonurethritis cases each were required. Prevalence of infections was compared between urethritis and no urethritis and also compared within urethritis cases for the same infection, using χ^2^ and McNemar test, respectively.

### Sequence Type Assignment and Phylogeny Construction

MG191 sequence type (ST) type was assigned on the basis of previously described types [15–17] numbered 1–80. Maximum likelihood phylogeny was reconstructed from ClustalW alignments using RAxML v7.3.2 using a generalized time reversible (GTR) model of nucleotide substitution with gamma model of rate heterogeneity [18]. Branch support values were generated from 1000 bootstrap replicates.

## RESULTS

### Prevalence of Infection

217 men were recruited, 18 of whom were men who have sex with men (MSM). One hundred ten of 217 had any urethritis by study definition and 102 of 217 had NGU. The prevalence of *C. trachomatis* and *M. genitalium* were similar in NGU cases and both significantly higher than in those with no urethritis (Table 1). The prevalence of both *M. genitalium* and *C. trachomatis* among MSM and heterosexual men with NGU was similar (data not shown). The clinic database was checked for all male attendees during the exact period of the study. Of 184 men diagnosed with NGU, 14 were diagnosed with *C. trachomatis*, giving a prevalence of 7.6% (95% confidence interval [CI], 4.4%–12.7%), lower than in our study. This may have reflected a different threshold for diagnosing NGU in the general clinic compared to our study criteria.

**Table 1.** Prevalence of *Neisseria gonorrhoeae*, *Chlamydia trachomatis*, *Mycoplasma genitalium*, and *Trichomonas vaginalis* for No Urethritis, Gonococcal Urethritis, Nongonococcal Urethritis (NGU), and Nonchlamydial NGU

Organism	No Urethritis	All Urethritis	*P* Value^a^ for All Urethritis	Nongonococcal Urethritis (NGU)	*P* Value^a^ for NGU	NCNGU	*P* Value^a^ for NCNGU
*Neisseria gonorrhoeae*	2/107; 1.9% (.0–4.5)	7/109; 6.4% (1.8–11.0)	.17	NA	…	NA	…
*Chlamydia trachomatis*	2/107; 1.9% (.0–4.5)	17/109; 15.6% (10.0–24.0)	.0001	15/102; 14.7% (7.8–21.6)	.0007	NA	…
*Mycoplasma genitalium*	5/107; 4.7% (.7–8.7)	17/110; 15.5% (8.7–22.3)	.0085	17/102; 16.7% (9.5–24.0)	.0047	17/87; 19.5% (11.2–27.8)	.0012
*Trichomonas vaginalis*	0/107 0%	2/110;1.8% (0–4.3)	.49	2/102; 2.0% (0–4.7)	.24	2/87; 2.3% (0%–5.0%)	.46
*P* value, Ct vs Mg^b^		1		.86			

Data in parentheses represent the 95% confidence intervals.

Abbreviations: Ct, *Chlamydia trachomatis*; Mg, *Mycoplasma genitalium*; NA, not applicable; NCNGU, nonchlamydial nongonococcal urethritis; NGU, nongonococcal urethritis.

^a^ *P* values represent comparisons with no urethritis.

^b^ *P* values are for comparisons between *C. trachomatis* and *M. genitalium* prevalence.

### Genotypic Resistance Markers

Genotypic resistance data were available for both the macrolide and fluoroquinolone antibiotics for all 22 *M. genitalium–*positive clinical samples (Table 2). Macrolide resistance was detected in 9 samples (41%; [95% CI, 20%–62%]). A single sample, that is, 4.5% (95% CI, 1%–21%), possessed a mutation in the QRDR of *parC* associated with fluoroquinolone resistance, and this genotype possessed no genotypic resistance markers of macrolide antibiotics. The *parC* sequence of the isolate containing a SNP within the QRDR and the sequences of domain V of the 23S rRNA gene have been deposited within GenBank under the nucleotide accession numbers HF947096 and HF572928–HF572950, respectively.

**Table 2.** Clinical, Demographic, and Genotypic Characteristics of Patients With *Mycoplasma genitalium* Infection

ID	Sexual Orientation	Diagnosis	Treatment	Antibiotics in Last 6 mo	Follow-up	*mgpB* SNP Type^a^	*MG309* vntr Copy Number	23S rRNA Mutation	Mutant Fluoroquinolone QRDR: Amino Acid Change^b^	Major Cluster *mgpB*^c^	Major Cluster Dual^c^
170	MSW	NGU	Doxycycline	ND	Nil	A	15	A2058G	WT	A	A
202	MSW	NGU	Doxycycline	ND	ND	44	Nontypeable	A2059C	WT	A	
11	MSW	Nil	Nil	ND	1 wk^d^	3	9	WT	WT	A	A
227	MSW	Nil	Nil	ND	1 mo^e^	3	11	A2059G	WT	A	A
107	MSW	NGU	Azithromycin 1 g	ND	5 mo^f^	E	15	WT	*parC:* S83R (80)	A	A
141	MSW	NGU	Azithromycin 1 g	ND	Nil	4	9	A2058G	WT	A	A
184	MSW	Nil	Nil	ND	Nil	J	8	A2059G	WT	A	A
183	MSW	Nil	Nil	Ceftriaxone	Nil	4	10	A2058G	WT	A	A
219	MSW	NGU-HSV	Doxycycline	ND	Nil	I	10	WT	WT	A	
189	MSW	NGU	Doxycycline	None	Nil	H	Nontypeable	WT	WT	A	
43	MSW	NGU	Doxycycline	ND	Nil	3	15	A2058G	WT	A	A
22	MSW	NGU	Doxycycline	Doxycycline	1 mo^g^	B	13	WT	WT	A	
174	MSM	NGU	Doxycycline	None	Nil	G	13	WT	WT	B	B
162	MSW	NGU	Doxycycline	ND	Nil	C	14	WT	WT	B	B
197	MSW	NGU	Azithromycin 1 g	Azithromycin 1 g	Nil	24	10	A2058G	WT	B	B
51	MSW	NGU	Doxycycline	ND	2 mo^h^	C	13	WT	WT	B	B
108	MSW	NGU	Azithromycin 1 g	ND	Nil	8	9	WT	WT	B	B
111	MSW	NGU	Doxycycline	ND	Nil	F	9	WT	WT	B	B
218	MSW	NGU	Doxycycline	ND	6 wk^i^	23	19	WT	WT	B	B
88	MSW	NGU	Azithromycin 1 g	ND	Nil	21	8	A2059G	WT	B	B
116	MSW	Nil	Nil	ND	Nil	2	11	WT	WT	B	B
72	MSW	NGU	Doxycycline	ND	Nil	D	10	WT	WT	B	B

Abbreviations: HSV, herpes simplex virus; MSW, men who have sex with women; ND, not documented; NGU, nongonococcal urethritis; Nil, no follow-up attendance; QRDR, quinolone resistance–determining region; rRNA, ribosomal RNA; SNP, single-nucleotide polymorphism; WT, wild type.

^a^ Sources: [8, 14, 15].

^b^ Parentheses represent the codon of *Escherichia coli* K12.

^c^ Both *mgpB* and dual sequence type phylogenetic analysis produced the same two major clusters as presented in Figure 1.

^d^ Persistent symptoms; doxycycline given.

^e^ Urethral discharge, nil found.

^f^ *Chlamydia trachomatis* diagnosed–asymptomatic.

^g^ Persistent symptoms; azithromycin (2 g) given over 5 days.

^h^ Persistent symptoms for 2 months; azithromycin (2 g) over 5 days and metronidazole given.

^i^ Persistent symptoms; doxycycline and metronidazole given.

There were no obvious clinical associations with antibiotic resistance. Macrolide-associated resistance markers were carried in 6 of 17 and 3 of 5 of those with NGU and no urethritis, respectively.

In 1 of these patients, carrying *A2058G*, the clinical notes indicated reception of single-dose azithromycin for a prior episode of NGU some months previously, raising the possibility of either treatment failure or selection of macrolide resistance from the previous episode. However, none of the 6 cases with NGU and macrolide resistance, 2 of whom were treated with doxycycline and 4 with single-dose azithromycin, returned to the clinic in the following 6 months with episodes of persistent or recurrent urethritis.

### Relatedness of *M. genitalium*

All 22 *M. genitalium* DNAs were successfully sequence typed by the MG191 SNP typing system. Eighteen of 22 were successfully typed using MG-309-STR. Table 2 displays MG191 (mgpB) and MG-309-STR types together with 23S rRNA type. Eleven of 22 *M. genitalium* infections were caused by 8 STs identical to MG191 alleles in the literature [14–16], designated ST 2, 3, 4, 8, 21, 23, 24, and 44. The remaining 11 sequence types exhibited novel MG191 alleles which we have designated as Novel STs A–J (Table 2). Sequences used for typing were deposited in GenBank under accession numbers KC445139 to KC445161 for the *mgpB* locus and KC445162 to KC445182 for the *MG309* locus.

**Figure 1.**
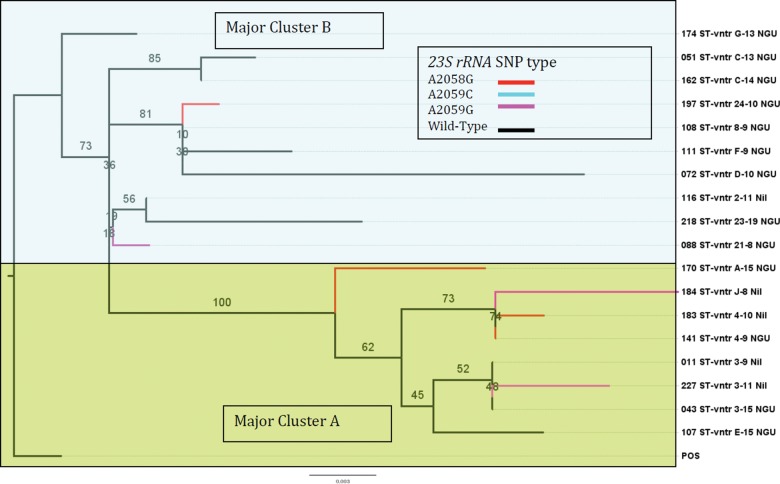
Phylogenetic tree of 18 of 22 dual locus sequence types of *Mycoplasma genitalium* detected from symptomatic men attending clinic. The tree was constructed only from samples in which both *MgpB* and *MG309* sequences were available using the *mgpB* sequence type combined with *MG309* vntr sequence (dual locus sequence type). Tips are colored by 23S rRNA single-nucleotide polymorphism type. Branch tips show identity number followed by the abbreviations ST, mgpB sequence type; vntr, MG309 sequence type repeat, and clinical syndrome; NGU, nongonococcal urethritis; Nil, no clinical syndrome. POS indicates positive control. The numbers along the branches represent bootstrap values. Major clusters A and B (see text).

Successfully typed MG-309-STR sequences were combined end-to-end with their respective MG191 sequences to form a dual locus sequence type (DLST). Phylogenetic analysis revealed a tree of 18 DLSTs, divided clearly into 2 major clusters, which we designated Major Cluster A (MCA) and B (MCB) (Figure 1). A phylogenetic tree, constructed using only MG191 sequences on study sequence types, produced identical assignments into these major clusters with poorer bootstrap values (data not shown). We therefore assigned the 4 sequence types, in which MG-309-STR had failed, to these major clusters accordingly (Table 2). Seven of 12 MCA types exhibited genotypic resistant mutations (4 *A2058G*, 2 *A2059G*, and 1 *A2059C*), compared with 2 of 10 in MCB (*A2058G* and *A2059G*) (Table 2 and Figure 1). We determined the phylogenetic relationship of study *M. genitalium* STs with 80 other previously typed strains [15–18] from Southern Europe and North Africa, which were typed using MG191-ST alone (Supplementary Figure). The tree suggests that our study *M. genitalium* STs, including those carrying 23S rRNA mutants, are distributed widely in this group of STs.

## DISCUSSION

*Mycoplasma genitalium* was a frequent but undiagnosed cause of NGU, as prevalent as *C. trachomatis* in our symptomatic, mainly heterosexual male cohort. In addition, >40% of *M. genitalium* infections had genotypic resistance to macrolide antibiotics, one of the recommended first-line options for NGU. A single patient, approximately 5% of the cohort, was infected with a strain demonstrating genotypic resistance to fluoroquinolones. Patients with *M. genitalium* infection, with or without genotypic resistance, separated into distinct genotypic clusters and were also related to a diverse population of international control sequence types. This indicated that our findings, rather than being a local clonal phenomenon that could have biased the data, were more likely to represent established infection and resistance rates in this patient population as a whole.

This also complements evidence that selection of macrolide-resistant *M. genitalium* during suboptimal treatment [19] contributes to cases of treatment failure rather than ongoing horizontal transmission of resistant bacterial strains. Overall, our findings indicate that there is a need to provide routine testing for *M. genitalium* in those presenting with symptoms of urethritis and perhaps cervicitis and that first-line recommended antimicrobial options for treating these syndromes need reappraisal [20, 21].

These treatment options will need to take into account the need to have effective regimens against both *C. trachomatis* and *M. genitalium* and should question the value of recommending either doxycycline or single-dose azithromycin alone. Whereas extended-dose azithromycin for NGU may be more effective in preventing the development of macrolide resistance and treatment failure, the in vitro and clinical data suggest that it will not work for those carrying 23s rRNA resistance-associated SNPs [19]. Future effective NGU regimens are likely to include fluoroquinolones. Solitary *parC* mutations, like the one documented in this study, have been reported in cases of fluoroquinolone treatment failure of *M. genitalium* [22, 23], and worryingly, there already exist reports of moxifloxacin treatment failure in *M. genitalium*–associated persistent NGU [24]. Collectively, these findings point to the need to evaluate effectiveness of new treatment strategies, including combination therapies and novel antimicrobials.

Effective management can be further guided by providing routine testing for *M. genitalium* infection [25], and tests of cure following therapy. The prospect of rapid and accurate tests for infections that include validated genetic markers of resistance [26, 27] will allow for appropriately targeted treatment and reduce need for complex regimens. Current rapid test platforms in development, including those recently approved for *C. trachomatis* and *Neisseria gonorrhoeae*, offer potential for such resistance tests to be realized [28].

The prevalence of *M. genitalium* in NGU in our study was perhaps at the lower end of the range of that described in previous work of 15%–25% [3]. Recent reports in South Africa mirror our findings of higher rates of *M. genitalium* infection relative to *C. trachomatis* [29], and importantly, comparable rates of macrolide resistance and emerging evidence of resistance to resistance to fluoroquinolones were reported in Sydney, Australia [30], suggesting that the problem may be globally widespread.

Although this study is limited by being based in a single clinic as well as conducted over a relatively short period of time, we addressed this by assessing the genetic diversity of *M. genitalium* within our cohort, utilizing a typing system with the discriminatory power to be useful in the study of sexual networks [14]. This robustly revealed 2 major clusters of *M. genitalium*, both of which contained drug-resistant mutations. Each cluster itself contained different MG191 STs, many of which had been previously identified. The MG191 typing system alone is less discriminatory and more useful to describe broad epidemiological STs [14]. Using this system, we showed that most STs within each cluster were not closely related as they were distributed broadly among international genotypes also typed using MG191.

In conclusion, the prevalence of macrolide-resistant *M. genitalium* in this population is much higher than previously thought and *M. genitalium* is detected as frequently as *C. trachomatis* in cases of NGU. Current testing and treatment guidelines should be modified to incorporate these findings.

## Supplementary Data

Supplementary materials are available at *Clinical Infectious Diseases* online (http://cid.oxfordjournals.org). Supplementary materials consist of data provided by the author that are published to benefit the reader. The posted materials are not copyedited. The contents of all supplementary data are the sole responsibility of the authors. Questions or messages regarding errors should be addressed to the author.

Supplementary Data
