# Supplementary material for: High Prevalence of Antibiotic-Resistant Mycoplasma genitalium in Nongonococcal Urethritis: The Need for Routine Testing and the Inadequacy of Current Treatment Options
Source: Clin Infect Dis. 2013 Nov 26;58(5):631–7. doi: 10.1093/cid/cit752 (PMC3922211; doi:10.1093/cid/cit752)
Supplement: Supplementary Data [file supp_58_5_631__index.html]

High prevalence of antibiotic resistant Mycoplasma genitalium in non-gonococcal urethritis: the need for routine testing and the inadequacy of current treatment options — High Prevalence of Antibiotic-Resistant Mycoplasma genitalium in Nongonococcal Urethritis: The Need for Routine Testing and the Inadequacy of Current Treatment Options — High Prevalence of Antibiotic-Resistant Mycoplasma genitalium in Nongonococcal Urethritis: The Need for Routine Testing and the Inadequacy of Current Treatment Options — Supplementary Data 

# High Prevalence of Antibiotic-Resistant *Mycoplasma genitalium* in Nongonococcal Urethritis: The Need for Routine Testing and the Inadequacy of Current Treatment Options

## Supplementary Data

Supplementary Data

**Files in this Data Supplement:**

- Supplementary Data - Doc file
