# Supplementary material for: High Prevalence of Antibiotic-Resistant Mycoplasma genitalium in Nongonococcal Urethritis: The Need for Routine Testing and the Inadequacy of Current Treatment Options
Source: Clin Infect Dis. 2013 Nov 26;58(5):631–7. doi: 10.1093/cid/cit752 (PMC3922211; doi:10.1093/cid/cit752)
Supplement: Supplementary Data [file supp_cit752_cit752supp.doc]

**Supplement for: High prevalence of antibiotic resistant *Mycoplasma genitalium* in non-gonococcal urethritis: the need for routine testing and the inadequacy of current treatment options. Marcus J Pond, Achyuta V Nori, Adam A Witney, Rose C Lopeman; Philip D Butcher, Syed Tariq Sadiq**

**Methods**

*Sample processing and pathogen detection*. Two mL of the FVU were aliquoted for routine NAAT for *N.gonorrhoeae* and *C.trachomatis* using the Becton Dickinson Viper system (Becton Dickinson, Oxford, United Kingdom) within 24 hours of sample submission. The residual of the FVU sample was centrifuged at 5000xg for 30 minutes and the urinary pellet re-suspended in 500µl of urine supernatant and stored at -70ºC. Urine pellets were retrieved and defrosted whilst spinning at 5000xg for 5 minutes at 4ºC. Total DNA was extracted using the FastDNA 2mL SPIN Kit for Soil as per manufacturer’s protocol (MP Biomedicals) and eluted DNA stored at -20 ºC until required.

Endpoint real time PCR was used for the detection of *M.genitalium* and *T.vaginalis* using primers as detailed by Jensen et al^1^ and Pillay et al^2^ respectively, with the following modifications. Reactions were carried out at a final volume of 20µl using QuantiFast Pathogen +IC mastermix (Qiagen, Crawley, United Kingdom) and 2µl of extracted target DNA. Reactions were performed in triplicate upon a Bio-rad CFX thermal cycler instrument. The median volume of urine used for the *M. genitalium* PCR assay was 23.5mLs (IQR 13.5 – 27.5).

*Sequencing of the genetic regions associated with resistance to macrolide and fluoroquinolone antibiotics.* For macrolide associated resistance, the DNA extracts from samples that were positive for *M.genitalium* endpoint PCR were investigated for presence of 23S rRNA mutations as described in the methodology described by Jensen et al.^3^ In order to detect genotypic resistance to fluoroquinlones, PCR primer pairs were designed to amplify quinolone resistance determining regions (QRDR) of the *gyrA*, *gyrB* and *parC* genes of *M.genitalium*. PCR primers used for sequencing QRDRs in this study are detailed in Table 1. PCR reactions were carried out in 50µl final reaction volumes using Platinum PCR supermix (Invitrogen, Paisley, United Kingdom) with primers at a final concentration of 0·4mmol/L of each primer. Thermal cycling was performed using a GS-1 thermocycler (G-Storm, Somerton, United Kingdom). The following cycling conditions were used: initial denaturation at 95ºC for 2 minutes followed by 40 cycles of 15 seconds at 95ºC, 60 seconds at 60ºC and 30 seconds at 72ºC. The PCR products were subject to a final extension at 72ºC for 30 seconds. Amplicons were purified using a MinElute Reaction Cleanup Kit (Qiagen, Crawley, United Kingdom) and sequenced bidirectionally using Big Dye terminator v3·1 (Applied Biosytems,Warrington, UK). The chromatogram files were quality trimmed using FinchTV v1·4 (Geospiza, Seattle, USA) were trimmed for quality (PHRED quality values >20) and the remaining sequence used for further analysis. Processed read pair sequence files were combined and assembled into contigs using the CAP3 program^4^ and compared to the *M.genitalium* G37 23S rRNA sequence (G37T;ATCC 33530T) using ClustalW (European Bioinformatics Institute, Cambridge, UK).

*M.genitalium typing*: Two genotyping techniques were utilized to assess the diversity of *M.genitalium* strains within our population. We used the previously described MG191 (*mgpB*) SNP typing method.^5^ This typing system assigns alleles to strains on the basis of sequence comparison to previously documented sequence types and can be used to provide an estimate of the overall diversity of strains present. Following this we combined MG191 data with a MG309 variable number tandem analysis (MG-309-STR).^6^ This methodology relies upon the enumeration of tri- or di- nucleotide repeat units occurring within the MG309 gene of *M.genitalium* and the numbers of copies identified and, when combined with *mgpB* typing, can be used to further assess the relatedness of strains.

PCR amplification and sequencing of the MG191 locus and MG309 variable number tandem repeat analysis was performed as described^5,6^ with the following changes: PCR amplifications were performed using Platinum PCR supermix (Invitrogen, Paisley, UK). Reactions contained 46µl of reaction buffer, 0·4 pmol of each primer (Operon Biotechnologies, Cologne, Germany) and 2µl of extracted DNA. Tandem repeats present within the MG309 variable region were detected using RepeatMasker software.^7^ PCR amplification conditions and techniques for visualization and sequencing of PCR products were the same as those described in the analysis genotypic resistance mutations.

**Supplement Table: PCR primers used for sequencing QRDRs**

| **Primer name** | **Sequence** |
| --- | --- |
| MG_gyrA_F | 5’-cctgatgctagagatggacttaaa-3’ |
| MG_gyrA_R | 5’-aagttctgctgcaagtttagataat-3’ |
| MG_gyrB_F | 5’-ttgtacaaccagagatccttcg-3’ |
| MG_gyrB_R | 5’-gtgggggttgagcaataaaa-3’ |
| MG_parC_F | 5’-gtgctgttggggagatcat-3’ |
| MG_parC_R | 5’-ccatggatagaaacagttgttca-3’ |

**Supplement Figure. Phylogenetic relationship of 22 *M.genitalium* strains from the study with 80 other previously typed strains^4,5,7^ using the MG191-ST alone.**

The tips of the tree in this figure are labeled by sample identification, assigned sequence-type and coloured by resistance associated mutations detected. The tree is rooted with MG191 sequence from the unrelated *M. genitalium* reference strain G37^T^ (ATCC 33530^T^
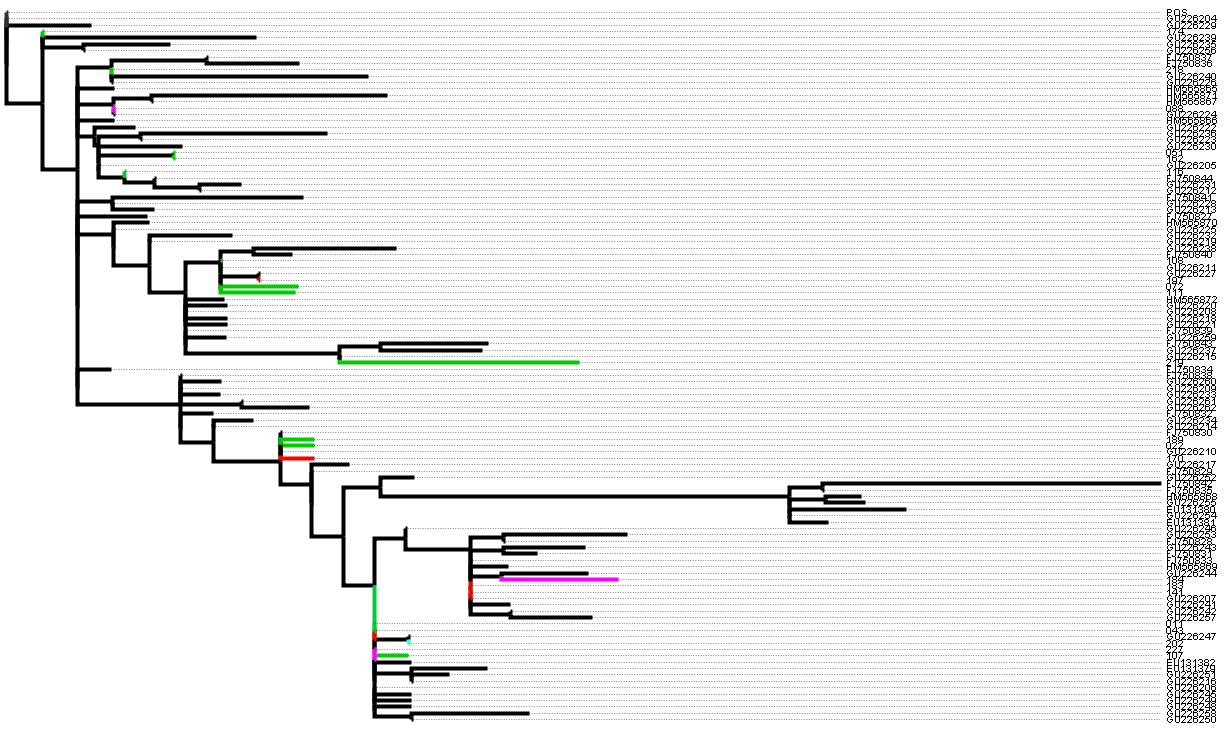
: “POS”).

Study *23S rRNA* types

A2058G

A2059C

A2059G

Wild-Type

Reference Samples

**References**

1. Jensen JS, Bjornelius E, Dohn B, Lidbrink P. Use of TaqMan 5' nuclease real-time PCR for quantitative detection of Mycoplasma genitalium DNA in males with and without urethritis who were attendees at a sexually transmitted disease clinic. J Clin Microbiol 2004; 42(2):683-692.

2. Pillay A, Radebe F, Fehler G, Htun Y, Ballard RC. Comparison of a TaqMan-based real-time polymerase chain reaction with conventional tests for the detection of *Trichomonas vaginalis*. Sex Transm Infect 2007; 83(2):126-129.

3. Jensen JS, Bradshaw C, Tabrizi S, Fairley C, Hamasuna R. Azithromycin treatment failure in *Mycoplasma genitalium*-positive patients with nongonococcal urethritis is associated with induced macrolide resistance. Clin Infect Dis. 2008; 47:1546-43.

4. Huang X, Madan A. CAP3: A DNA sequence assembly program. Genome Res. 1999 Sep;9(9):868-77.

5. [Hjorth SV](http://www.ncbi.nlm.nih.gov/pubmed?term=Hjorth SV%5BAuthor%5D&cauthor=true&cauthor_uid=16757601), [Björnelius E](http://www.ncbi.nlm.nih.gov/pubmed?term=Björnelius E%5BAuthor%5D&cauthor=true&cauthor_uid=16757601), [Lidbrink P](http://www.ncbi.nlm.nih.gov/pubmed?term=Lidbrink P%5BAuthor%5D&cauthor=true&cauthor_uid=16757601), et al. Sequence-based typing of *Mycoplasma genitalium* reveals sexual transmission. Clin Microbiol. 2006 Jun;44(6):2078-83.

6. [Cazanave C](http://www.ncbi.nlm.nih.gov/pubmed?term=Cazanave C%5BAuthor%5D&cauthor=true&cauthor_uid=22160316), [Charron A](http://www.ncbi.nlm.nih.gov/pubmed?term=Charron A%5BAuthor%5D&cauthor=true&cauthor_uid=22160316), [Renaudin H](http://www.ncbi.nlm.nih.gov/pubmed?term=Renaudin H%5BAuthor%5D&cauthor=true&cauthor_uid=22160316), [Bébéar C](http://www.ncbi.nlm.nih.gov/pubmed?term=Bébéar C%5BAuthor%5D&cauthor=true&cauthor_uid=22160316). Method comparison for molecular typing of French and Tunisian Mycoplasma genitalium-positive specimens. J Med Microbiol. 2012 Apr;61(Pt 4):500-6. Epub 2011 Dec 8.

7. A.F.A. Smit, R. Hubley & P. Green RepeatMasker at http://repeatmasker.org).
